# Evaluation of the repeatability of corneal epithelial thickness mapping in healthy and keratoconic eyes with two spectral domain optical coherence tomography

**DOI:** 10.1016/j.optom.2025.100535

**Published:** 2025-02-05

**Authors:** Branka Samolov, Stephanie van de Moosdijk, Abinaya Priya Venkataraman, Alberto Domínguez-Vicent

**Affiliations:** a Division of eye and vision, Karolinska Institutet, Solna, Sweden; b St Erik Eye Hospital, Solna, Sweden

**Keywords:** Keratoconus, Epithelial thickness, Anterior segment optical coherence tomography oct, repeatability, precision

## Abstract

**Purpose:**

To evaluate the repeatability of corneal epithelial thickness measurements using anterior segment optical coherence tomography (AS-OCT) and a posterior segment OCT adapted with an anterior module, in subjects with keratoconus and healthy controls.

**Methods:**

A spectral domain AS-OCT (MS-39) and a posterior segment OCT (HS-100) with ASA-1 adaptor were used to measure the corneal epithelial thickness in healthy and keratoconic eyes. Three measurements per participant were taken, and the repeatability was described using the repeatability limit (Rlim), calculated from the within-subject standard deviation.

**Results:**

81 eyes of 81 controls and 80 eyes of 52 keratoconus subjects (43 % cross-linking, and 13 % contact lens users) were included. For the MS-39, the central sector showed the best repeatability for both groups, with Rlim never exceeding 5 μm in any sector. For the HS-100, the best repeatability was obtained for the central sector, with the Rlim never exceeding 7 μm in any of the sectors for the control group and all but one (outer-inferior) in the keratoconus group. The Rlim for the keratoconus group varied <1 μm between contact users/non-users or between eyes with/without a history of CXL. Differences in Rlim were larger than 2 μm in the peripheral horizontal sectors between each sub-group with the HS-100.

**Conclusions:**

Both OCTs showed good epithelial thickness measurement repeatability in all groups, though the repeatability of the HS-100 was mildly lower for keratoconic eyes. Contact lens use and crosslinking history did not affect repeatability. These OCTs effectively measure epithelial thickness in keratoconus patients, which could be helpful in monitoring keratoconus progression.

## Introduction

Corneal imaging is an essential tool in modern eye care. Initially, the curvature of the anterior corneal surface constituted the ground for the estimation of corneal optical properties and diagnosis of ectatic disorders like keratoconus. Gradually the importance of the posterior corneal curvature as well as its thickness gained significance.^1^, ^2^, ^3^ In recent years, new imaging devices allowing fast and high-resolution imaging of the entire anterior segment, cornea included started to emerge. The main advantages of the new generation Anterior Segment Optical Coherence Tomography (AS-OCT) are their superior acquisition speed, improved optical resolution (axial and transversal), as well as scanning depth and field of view.^4^ High-resolution imaging of the cornea with AS-OCT provides thickness measurements individually for epithelium and stroma individually.^5^^,^ ^6^ Measuring corneal epithelial thickness adds information that could be of value in early diagnosis, progression evaluation and maybe even for a more complete understanding of the pathophysiology of this disease.^7^ It has been shown that changes in the corneal epithelial thickness could be a sensitive tool for early keratoconus detection as well as for the assessment of the disease progression.^8^

Early diagnosis with subsequent corneal crosslinking (CXL) can slow-down or stop keratoconus progression.^9^ Identifying subclinical keratoconus is extremely important in refractive surgery screening to avoid iatrogenic ectasia.^10^^,^^11^ Anterior and posterior corneal topography combined with total cornea thickness measurements and localisation of the thinnest area are the parameters used commonly for diagnosing and staging of corneal ectasias. Recently, epithelial thickness is also added as an additional parameter and is shown to be useful for both early diagnosis and grading of keratoconus.^12^, ^13^, ^14^

Precise measurements of the epithelial thickness are therefore important for keratoconus diagnosis and follow-up. AS-OCTs have high axial resolution and studies have shown that these instruments provide repeatable epithelial measurements both in central and peripheral cornea.^15^^,^^16^ With an additional lens (anterior segment module), posterior segments OCTs can also be used to measure corneal parameters including epithelial thickness^15^^,^^17^^,^^18^ and previous studies have shown that these modules can also produce precise epithelial thickness measurements in healthy corneas and in keratoconus. There is only limited information on how the precision of epithelial measurements with the anterior module in posterior segment OCTs compares to the precision of epithelial measurements with AS-OCTs.

This study aimed to evaluate the repeatability of epithelial and total corneal thickness measurements with an AS-OCT and a posterior segment OCT with an anterior segment module, in subjects with keratoconus and healthy controls.

## Methods

### Subjects

Eyes with and without keratoconus were included in this cross-sectional study. Individuals with a previous diagnosis of keratoconus were invited to participate in the study during their annual follow-up session at the Department of Anterior Segment (S:t Erik Eye Hospital, Eye Center of Excellence, Stockholm, Sweden). Subjects with a history of ocular surgeries other than corneal cross-linking were excluded from the study. The control group was recruited from the Optometry clinic (Eye Center of Excellence, Karolinska Institutet, Stockholm, Sweden), and no selection bias was considered as long as the subjects fulfill the following criteria. To be included int the control group, the subjects should not have any known ocular diseases or previous ocular surgery. Only subjects older than 18 years of age were included in this study.

The study was approved by the Regional Ethics Committee (Swedish Ethical Review Authority, Drn. 2021–03835) and was conducted following the tenets of the Declaration of Helsinki. Written informed consent was obtained from all subjects before their enrollment in the study.

### Instrumentation

Two different OCTs were analysed in this study:

1. MS-39 (Costruzione Strumenti Oftalmici, Firenze, Italy) combines a spectral domain AS-OCT and Placido disk corneal topographer. The OCT uses a wavelength of 845 nm, scanning an area of 16 × 7.5 mm resulting in 25 B-scans each of which consists of 1024 A-scans. The axial and transversal resolutions are 3.50 μm and 35 μm, respectively. The data from the Placido disk topography (wavelength of 635 nm) and OCT scans are merged to calculate corneal curvature (anterior and posterior surface) and thickness. Besides specific cornea measurements, the device provides analysis of the remaining structures of the anterior segment as well. Epithelial thickness measurements are divided into 9 sectors (Fig. 1, left panel). The thinnest epithelial and corneal measurements are obtained separately.

**Fig. 1:**
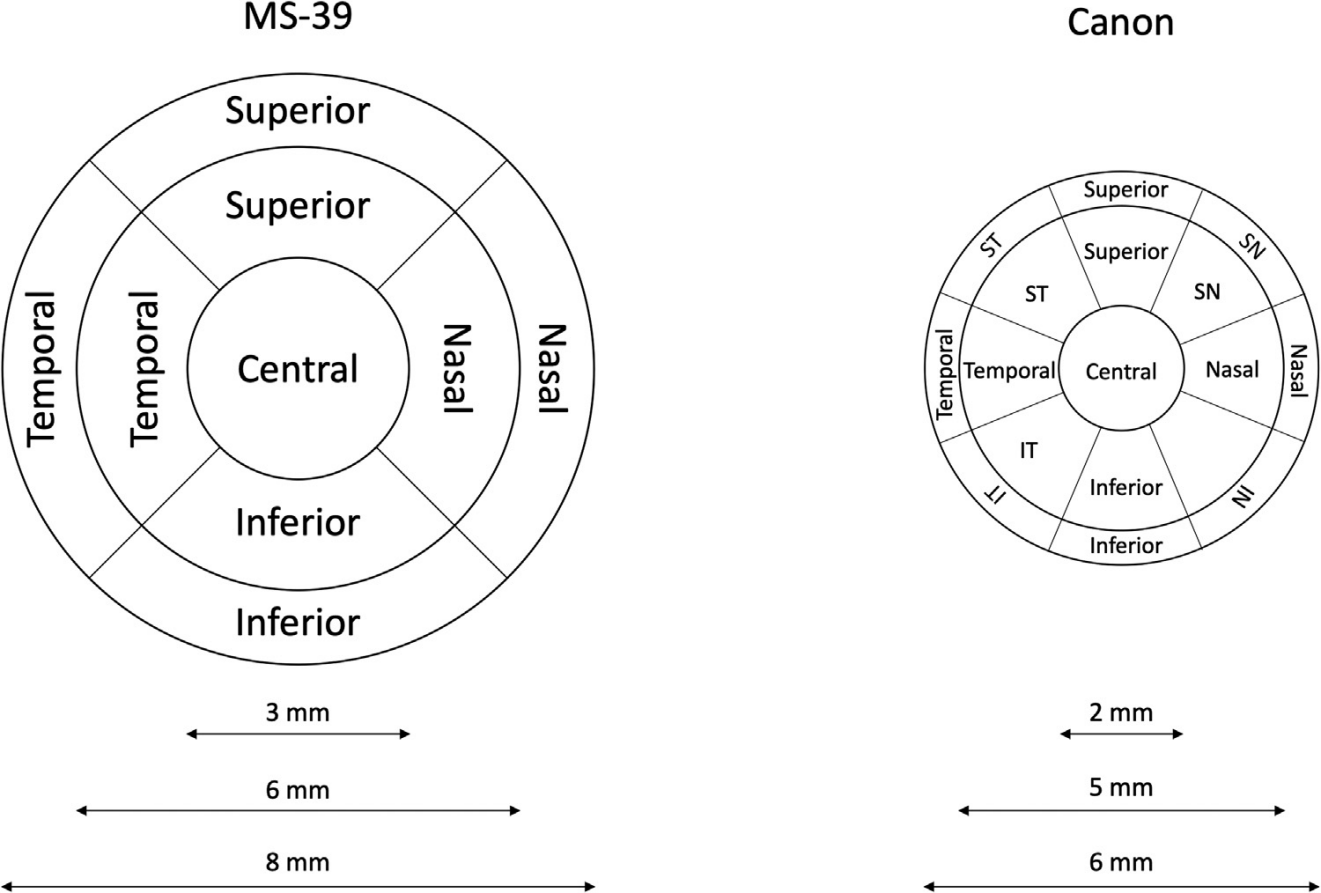
Schematic diagram of the epithelial maps used in each instrument.
2. OCT HS-100 (Canon Europe, Netherlands) is a spectral domain OCT. This posterior segment OCT has a wavelength of 855 nm, and it can be converted into an anterior segment OCT with the adaptor ASA-1. The scan area is 6 × 6 mm, containing 24 B-scans, each consisting of 1024 A-scans. The axial and transversal resolution without the ASA-1 adapter are 3 µm and 20 µm, respectively. The epithelial thicknesses are measured in 17 sectors (Fig. 1, right panel). In addition, also this device provides the thinnest cornea measurements.

As Fig. 1 shows, the thickness map instruments have different the thickness maps

### Procedure

The order of each instrument was randomized for each participant in both groups. 3 measurements were taken on each participant under repeatability conditions in a single session.^19^ Concretely, the environmental conditions were kept constant during data collection (lighting, temperature, etc.), and the same experienced examiner performed all measurements. Also, the subject was instructed to seat back and the instrument was decentered after each measurement. One eye from each participant in the control group was included randomly. In the keratoconus group, both eyes were measured, and only one eye was included randomly if the keratoconus grade was symmetric between the eyes. For evaluating symmetry, the C parameter (thinnest pachymetry) from the Belin ABCD keratoconus staging criteria.^20^ Within the keratoconus group, the participants were divided according to contact lens usage or history of CXL. Contact lens users were instructed to remove their lenses before the measurements were performed. From both instruments, the corneal epithelial thickness values were extracted and analysed.

### Statistical analysis

The descriptive statistics were calculated for the demographic parameters and the epithelial thickness. The repeatability of each instrument was analysed based on the standards adopted by the British Standards Institute and the International Organization for Standardization.^21^ The repeatability of each instrument was described in terms of within-subject standard deviation (S_w_) and repeatability limit (Rlim), calculated as $1.96\sqrt{2}\cdot S_{w}$. The Shapiro–Wilk test was used to check the normality distribution. As Fig. 1 shows, the thickness map of each instrument was different in size and design. For example, the diameter of the thickness map from the MS-39 is 8 mm, and the diameter from the HS-100 is 6mm. Thus, no agreement analysis was performed to compare the epithelial thickness from both instruments.

The required sample size (n) for this study was calculated based on the number of repeated measurements (m) and the confidence level (CL) for the Sw using the following formula $\frac{1.96}{\sqrt{2n(m-1)}}$ = CL.^21^ Considering a CL of 0.12 and 3 repeated measurements, a minimum of 67 eyes were required.

## Results

In total, 81 eyes of 81 controls and 80 eyes of 52 subjects with keratoconus were included in this study. The population demographics are summarized in Table 1.

**Table 1:** Population demographics.

Parameter		Control	Keratoconus	p-value
Number of eyes (subjects)		81 (81)	80 (52)	NA
Age (years)		30.8 ± 11.3 (19–63)	33.6 ± 10.01 (18–73)	0.10
Males:Females		22:59	44:8	<0.001
RE:LE		42:39	37:43	0.48
Eyes with cross linking		0	34	NA
CCT (microns)		539±32.66	494±38.94	<0.001
Anterior Ks at 3mm (Dioptres)		44.36±1.57	52.92±4.82	<0.001
Subjective refraction(Dioptres)	**M**	–1.20±2.62 (–13.25 to +4.00)	–0.89±2.20 (–12.00 to +1.75)	0.450
	**J0**	0.17±0.47 (–1.37 to +1.87)	–0.32±1.20 (–4.00 to +4.92)	<0.001
	**J45**	0.01±0.31 (–1.38 to +0.97)	–0.16±0.99 (–2.82 to +2.59)	0.14

Age and subjective refraction are expressed as mean ± standard deviation and range. Age is expressed in years, Anterior corneal power and subjective refraction is expressed in Dioptres. STD, standard deviation. RE, Right eye. LE, Left eye. M, Spherical equivalent. J0 and J45: Cylindrical vectorial components.

**Fig. 2:**
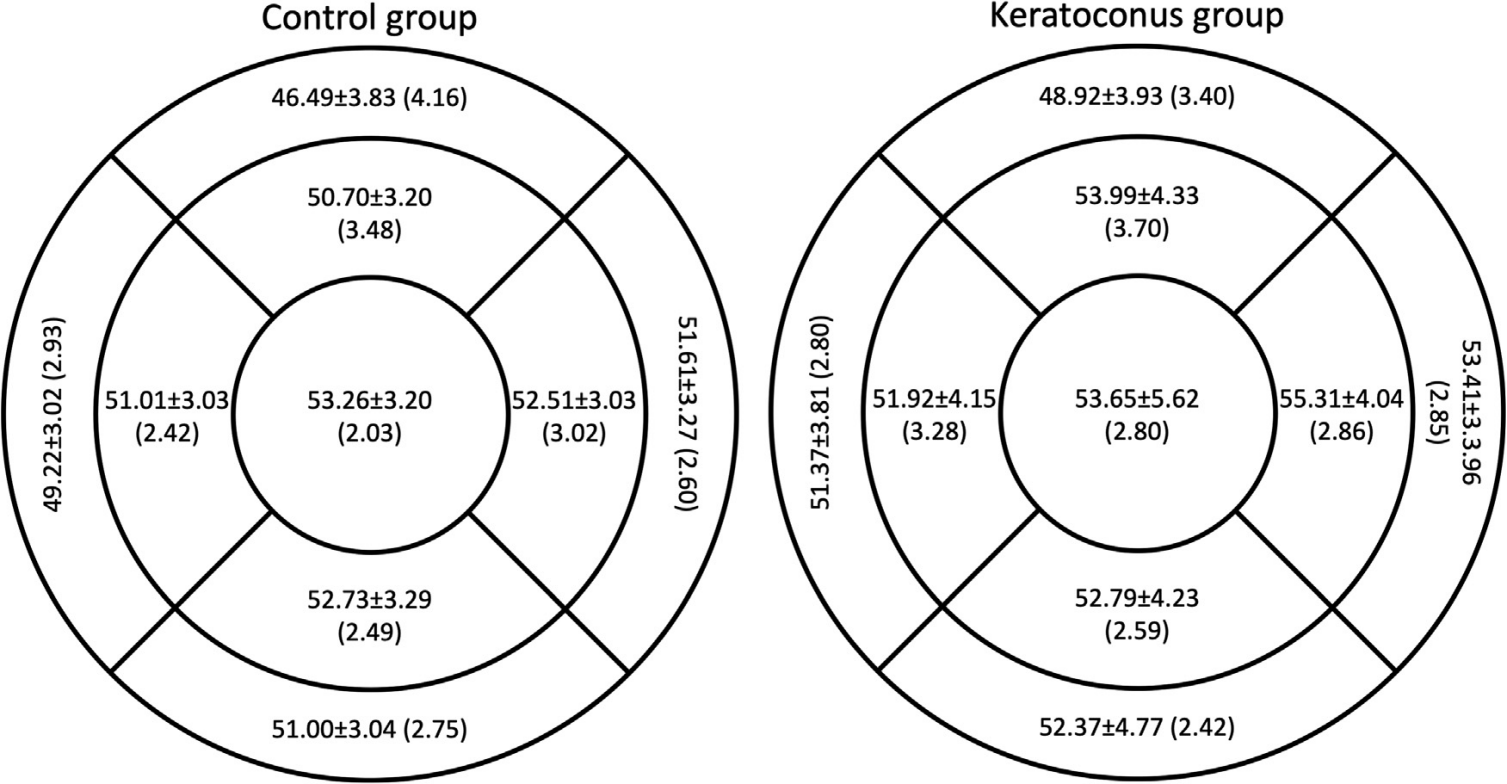
Average epithelial thickness and its corresponding repeatability limit in each sector for the MS-39. The orientation of the sectors corresponds to the right eye.

Fig. 2 shows the average epithelial thickness and Rlims obtained with the MS-39 for the control group (left panel) and the keratoconus group (right panel). The best repeatability was obtained in the central sector for both control and keratoconus groups, and the value was similar between both groups (2.03 μm and 2.80 μm, respectively). The Rlim never exceeded 5 μm in any of the sectors for both groups. Within the same population group, the Rlim did not vary >2 μm between all sectors. Comparing the Rlim between both groups for the same sector, the difference was lower than 1 μm.

**Fig. 3:**
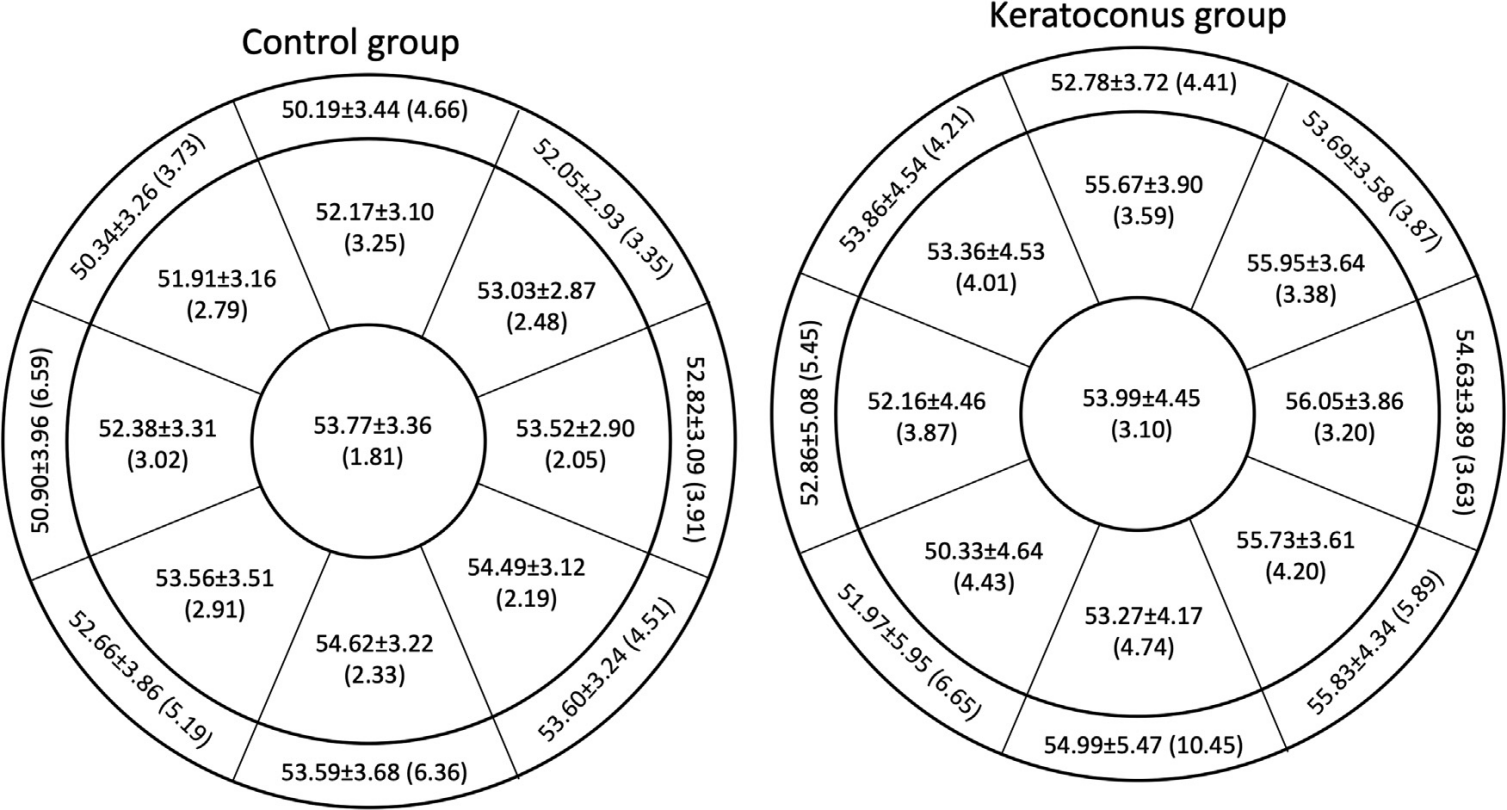
Average epithelial thickness and its corresponding repeatability limit in each sector for the HS-100. The orientation of the sectors corresponds to the right eye.

Fig. 3 shows the average epithelial thickness and Rlims values obtained with HS 100 for the control (left panel) and keratoconus (right panel) groups. The best repeatability was obtained for the central sector in both groups, but the values were different between the healthy and keratoconic groups (1.81 μm and 3.10 μm, respectively). The Rlim never exceeded 7 μm in any of the sectors for the control group and in all but one sector in the keratoconus group (outer inferior sector, the Rlim: 10.45 μm). For both groups, the Rlim values were more homogeneous for the inner sectors compared to the outer sectors. In the control group, the maximum difference in the Rlim was 1.20 μm within the inner sectors and 3.24 μm within the outer sectors. In the keratoconus group, the maximum difference in the Rlim was 1.54 μm within the inner sectors and 6.82 μm within the outer sectors. Comparing the Rlim values between the groups for the same sector, the Rlim is larger in the keratoconus group. The differences in the Rlim between the groups were small, except for the outer inferior sector (4.09 μm difference).

Table 2 shows the Rlim values for the keratoconus group, sub-grouped based on contact lens and history of CXL. For MS-39, the Rlim values did not wary more than a micron in any of the sectors between contact users and non-users as well as between eyes with and without a history of CXL. However, the differences in Rlim were larger than 2 μm in the peripheral horizontal sectors between each sub-group (CL users/no CL users, and CXL/no CXL) with the posterior segment OCT.

**Table 2:** Repeatability limit for each thickness sector map for contact lens users and non-contact lens users.

MS-39 Sectors	CL	No- CL	CXL	No-CXL
Central 3 mm	2.63	2.87	2.90	2.78
Nasal 3–6 mm	3.29	2.78	2.82	2.91
Temporal 3–6 mm	3.45	3.22	3.21	3.30
Superior 3–6 mm	2.97	3.80	3.92	3.49
Inferior 3–6 mm	2.51	2.60	2.56	2.60
Nasal 6–8 mm	2.71	2.86	2.53	3.03
Temporal 6–8 mm	3.15	2.73	2.33	3.09
Superior 6–8 mm	3.33	3.39	3.40	3.36
Inferior 6–8 mm	2.95	2.34	2.08	2.69

Canon HS 100 Sectors	CL users	Non- CL	CXL	No-CXL

Central 2 mm	2.17	3.24	3.22	3.00
Temporal 2–5 mm	3.84	3.87	4.16	3.63
Superior Temporal 2–5 mm	4.79	3.85	4.35	3.76
Superior 2–5 mm	2.47	3.75	3.45	3.66
Superior Nasal 2–5 mm	2.35	3.52	3.06	3.56
Nasal 2–5 mm	2.66	3.31	2.92	3.41
Inferior Nasal 2–5 mm	2.65	4.41	4.11	4.22
Inferior 2–5 mm	4.57	4.91	4.92	4.82
Inferior Temporal 2–5 mm	3.46	4.64	4.77	4.24
Temporal 5–6 mm	6.54	5.21	3.85	6.37
Superior Temporal 5–6 mm	3.70	4.27	4.72	3.77
Superior 5–6 mm	3.37	4.56	4.69	4.17
Superior Nasal 5–6 mm	3.45	3.95	3.28	4.26
Nasal 5–6 mm	3.19	3.70	3.78	3.50
Inferior Nasal 5–6 mm	8.51	5.24	6.35	5.54
Inferior 5–6 mm	6.01	11.02	10.44	10.36
Inferior Temporal 5–6 mm	4.49	7.00	5.15	7.58

**Fig. 4:**
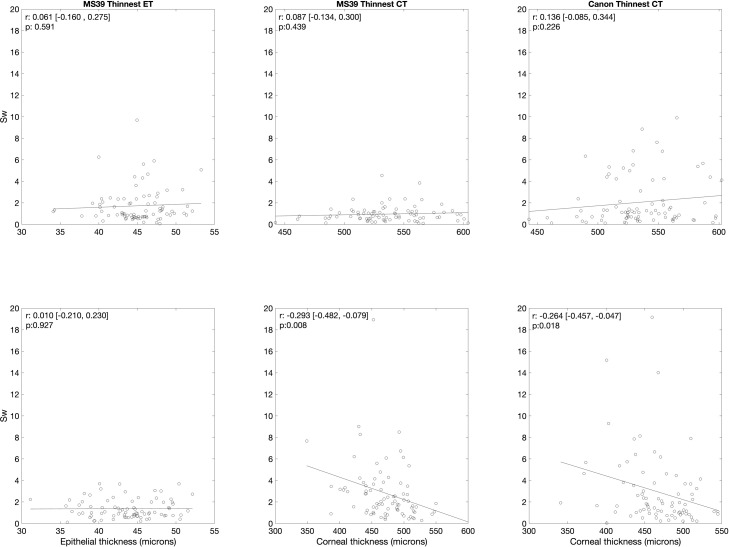
Correlation between the thinnest epithelial thickness measured with the MS-39, the thinnest corneal thickness measured with the MS-39, and the thinnest corneal thickness measured with the Canon OCT with their respective SW values. Correlation coefficient (r) and level of significance (p) are given on the top left corner of each plot. Values in the square brackets represent the lower and upper bounds for a 95 % confidence interval of r.

Fig. 4 shows the correlation between the thinnest epithelial thickness measured with the MS-39, the thinnest corneal thickness measured with the MS-39, and the thinnest corneal thickness measured with the Canon OCT with their respective Sw values for both groups. For the control group, the correlations between the three different metrics and their respective Sws were not statistically significant (*p* > 0.05). Regarding the keratoconus group, the was a weak negative correlation (*p* < 0.05) between the thinnest corneal thickness and their respective Sw for both instruments.

## Discussion

In this study, we evaluated the repeatability of corneal epithelial thickness measurements in subjects with keratoconus and controls, performed with two different OCTs: one anterior and one posterior segment OCT with an anterior segment module. The anterior segment OCT (MS-39) showed similar repeatability among the groups, whereas the posterior segment OCT adjusted for anterior segment measurements (HS-100) showed slightly better repeatability for the control group than the keratoconus group.

The epithelial thickness is of importance as it is affected in keratoconus and can be used both in the diagnosis, including subclinical stages of ectasia, as well as in follow-up.^14^^,^^22^^,^^23^ A detailed description of the anterior segment including corneal epithelial thickness mapping can be acquired by combining topographic and tomographic imaging. The anterior segment OCT evaluated in this study showed similar performance for both keratoconus and control group, where the Rlim was similar to the axial resolution of the instrument (3.6 μm). These results are similar to those obtained in previous studies assessing the repeatability of the epithelial thickness measured with anterior segment OCT, which reported similar repeatability values,^6^^,^^16^^,^^24^^,^^25^ even with instruments with an axial resolution of 10 μm.^6^^,^ ^24^ Concretely, the Rlim values obtained in these studies ranged from about 0.9 to about 7μm.

The converted OCT showed different repeatability results between the groups, although the differences were minimal from a clinical point of view. Also, this pattern confirms the results of previous studies using different convertible OCTs.^24^^,^^26^ This OCT showed lower repeatability values at the inferior sectors, which could be related to instrument limitations. In this study, there repeatability was slightly different between both devices, especially in the most outer sectors. This could be related to several factors such as instrument resolution, segmentation algorithm, instrument design or patient related artefacts. Unfortunately, direct performance comparisons between the tested instruments OCTs were not possible as the map designs available for clinical use are different and cover different corneal areas. It would be of interest to assess the repeatability of instruments with different designs or measurement principles in subjects with keratoconus at different stages.

Contact lens users were included in this study, and subjects were instructed to remove their lenses before the measurements were performed. It has been reported that the corneal epithelium is thinner in long-term contact lens-users (at least 6 months) of either soft or hard contact lenses.^27^ Thus, it seems evident that subjects who have been wearing contact lenses for a long time will have a thinner epithelium than those who are not lens users. Also, the thinner the epithelial layer the harder it can be to delineate it, which could be a methodological drawback in clinics as this will affect the layer segmentation. However, in the present study, the main objective was to assess the repeatability of the corneal epithelial thickness measured with two different instruments. We measured the subjects with both instruments at the same time, under the same conditions and our results showed that the repeatability was not worse in contact lens users for both instruments, thus confirming that the reliability was not affected by the contact lens use.

As Table 1 depicts, there was a statistically significant differences in the gender ratio between both population group. Concretely, there were double number of males in the keratoconic group, and about 7 times more women in the healthy control. As It has been reported, males and females have similar corneal diameter^28^ and thickness.^29^ Traditionally, it has been reported that the prevalence of keratoconus is larger in males,^30^^,^^31^ although recent studies have shown that gender is not a risk factor to develop keratoconus.^32^, ^33^, ^34^ Based on these later results, we could conclude that differences in the gender ratio between both population groups would not be a confounder factor.

Previous studies comparing the performance of spectral domain and swept source OCTs in measuring the epithelial thickness have shown similar results for both healthy control and keratoconus groups.^6^^,^^24^ For diagnostic and follow-up purposes, both OCT technologies can be used as the repeatability is similar. From the current repeatability measurements, the measurement tolerance ($MT=1.96\cdot \frac{Sw}{\sqrt{N}}$) can be calculated for both instruments for N number of measurements. Using the largest Sw value from the inner circle, where we would expect the early epithelial changes, the MTs for 1, 2 and 3 repeated measurements would be 2.6, 1.8 and 1.5 μm, respectively for measurements with MS-39. For measurements taken with the HS-100, the MTs for 1, 2 and 3 repeated measurements would be 3.4, 2.4 and 1.9 μm, respectively. Hence, depending on the accuracy needed for clinical management, what can be considered clinically acceptable or for research purposes, one can decide how many repeated measurements are needed.

Although the repeatability of the measurements did not show correlation with the epithelial thickness in general, the thinnest corneal thickness and the respective repeatability showed a weak negative correlation for both instruments. Even though the repeatability of the epithelial thickness measurements did not dependent on the actual epithelial thickness, these results show that higher the thinnest corneal thickness better the repeatability with both instruments. This could be because thinner layers might be harder to be segmented precisely, which will affect the instrument repeatability. In this study, the keratoconus group included both eyes with and without a history of CXL. It has been previously reported that the crosslinking does not have an impact on the repeatability of the epithelial thickness measurement,^15^ which also was corroborated by this study. However, it would be of interest to study this at different postoperative time points, which was not in the scope of this study. It would be good to assess the repeatability in eyes that had undergone cross-linking at different postoperative time courses and evaluate the influence of corneal haze. In this study, we could not assess the agreement between both instruments to measure the corneal epithelial thickness because the thickness map of each instrument was different in size and design. It would be advisable to have a standardized corneal thickness map across all devices.

In conclusion, both OCTs showed good epithelial thickness measurement repeatability in all groups, though the repeatability of the HS-100 was slightly lower for the keratoconic group. Contact lens use and crosslinking history did not affect repeatability. These OCTs effectively measure epithelial thickness in keratoconus patients, which could be helpful in monitoring keratoconus progression.

## Funding

10.13039/100010810Ögonfonden 2023.

## Conflict of interest

The authors declare no conflict of interest.
